# Dietary Models and Cardiovascular Risk Prevention in Pediatric Patients

**DOI:** 10.3390/nu15163664

**Published:** 2023-08-21

**Authors:** Maria Elena Capra, Delia Monopoli, Nicola Mattia Decarolis, Antonella Giudice, Brigida Stanyevic, Susanna Esposito, Giacomo Biasucci

**Affiliations:** 1 Pediatrics and Neonatology Unit, Guglielmo da Saliceto Hospital, 29121 Piacenza, Italy; m.capra@ausl.pc.it (M.E.C.); giacomo.biasucci@unipr.it (G.B.); 2 Società Italiana di Nutrizione Pediatrica, 20126 Milan, Italy; 3 Pediatric Clinic, Department of Medicine and Surgery, University Hospital of Parma, 43126 Parma, Italy; deliamonopoli@live.it (D.M.); nicolamattia.decarolis@unipr.it (N.M.D.); antonella.giud@gmail.com (A.G.); brigida.stanyevic@unipr.it (B.S.); 4 Department of Medicine and Surgery, University of Parma, 43126 Parma, Italy

**Keywords:** cardiovascular risk, dietary patterns, heart healthy diet, pediatrics, prevention

## Abstract

Nutritional intervention is worldwide recognized as a first step treatment for subjects with increased cardiovascular risk and it is of utmost importance especially for children and adolescents. Currently scientific evidence supports the role of dietary patterns instead of simple single nutrients or foods in cardiovascular risk prevention. Indeed, the American Heart Association dietary guidelines have expanded beyond nutrients to dietary pattern, that comprise not only single food items but also behavioral or cultural habits of specific populations. The aim of our narrative review is to analyze the most frequently adopted dietary patterns in children and adolescents and to evaluate their effect on cardiovascular risk factors and in cardiovascular risk prevention. Literature review showed that children cannot be considered as little adults: nutritional intervention must always grant adequate growth and neurodevelopment before reaching the proposed goals, therefore dietary patterns considered heart-healthy for adult subjects might not be suitable for pediatric patients. Mediterranean diet, DASH diet, Nordic diet and some plant-based diets seem to be the most promising dietary patterns in terms of cardiovascular health in the developmental age, even if further studies are needed to better standardize and analyze their effect on growing up individuals.

## 1. Introduction

**Figure 1:**
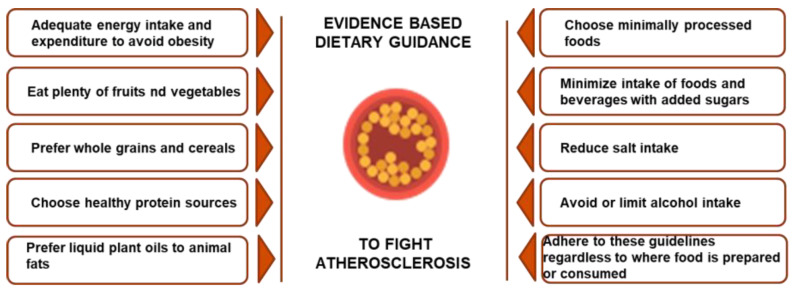
Heart-healthy diet criteria for adult subjects.

Nutritional intervention is a first step treatment for patients with increased cardiovascular (CVD) risk and it is of utmost importance especially for children and adolescents [1,2,3,4,5]. Currently, scientific evidence supports the role of dietary patterns instead of simple single nutrients in CVD prevention [6] and in the last decades, American Heart Association (AHA) dietary guidelines have expanded beyond nutrients to dietary pattern, that comprise not only single food items but also behavioral or cultural habits of specific populations [7,8].The most recent 2021 AHA Dietary Guidance analyzes a set of criteria for heart-healthy diets in adult subjects; these criteria promote cardiometabolic health and includes 10 features that pertain to food group guidance [9], as shown in Figure 1.

In a document published in 2023 [6], the authors identified popular dietary patterns commonly consumed in the United States and evaluated their adherence with the 2021 AHA Dietary Guidance. Mediterranean diet (MD), vegetarian diet and DASH diet resulted highly adherent to AHA Dietary Guidance, as well as vegan and low-fat diets, whereas Paleolithic diet was in the bottom tier, with a low adherence to AHA recommendations.

The aim of our narrative review is to analyze the most frequently adopted dietary patterns in children and adolescents and to evaluate their effect on cardiovascular risk factors and in cardiovascular risk prevention. Children cannot be considered as little adults: nutritional intervention must always grant adequate growth and neurodevelopment before reaching the proposed goals, therefore dietary patterns considered heart-healthy for adult subjects might not be suitable for pediatric patients [10].

The MEDLINE–PubMed database was searched to collect and select publications from 1990 to 2023. The search included randomized placebo-controlled trials, controlled clinical trials, double-blind, randomized controlled studies and systematic reviews. The following combinations of keywords were used: “heart-healthy diet” AND “nutrition” OR “dietary patterns” AND “Mediterranean” OR “Plant-based” OR “DASH” OR “Low-carbohydrate” OR “Ketogenic Diet” OR “Nordic Diet” OR “Paleolithic Diet” OR “High-Protein Diet” AND “children” OR “pediatric” OR “pediatric” OR “adolescent”. We also performed the manual search of the reference lists of the selected studies. The search was limited to English-language journals and full papers only.

## 2. Mediterranean Diet

MD is the main nutritional model referred to in International Guidelines as being effective in terms of CVD risk prevention [1,2,6]. The traditional MD is historically defined as the nutritional model mainly adopted in the olive tree-growing areas of the Mediterranean region up to the early 1960s. It is characterized by high daily intake of vegetables, fruits, pulses, and cereals (mainly in raw, unprocessed forms), by low intake of meat and meat-derived products, and low to moderate intake of dairy products. Other distinctive characteristics are a moderate/high fish intake and predominant use of unsaturated lipids, especially in the form of olive oil [11]. MD is one of the most studied dietary pattern. There is compelling evidence of its beneficial effects on health [11,12], enough to be declared Intangible Cultural Heritage of Humanity [11,13]. What is more, due to the great variety of foods proposed, its sustainability and adherence potentials are very high [14].

### 2.1. Mediterranean Diet and CVD Prevention in Adult Population

Fitó et al. analyzed variations of the MD pattern: the general MD included the use of olive oil in the MD + olive oil group, (≥4 tablespoon/day) for cooking and dressing, high use of fresh fruits (≥3 servings/day), legumes (≥3 servings/week), vegetables (≥2 servings/week), fish and seafood (≥3 servings/week), and walnuts (≥3 servings/week). In the MD + nuts group, nuts had to be consumed daily (30 g, composed of 15 g of walnuts, 7.5 g of almonds, and 7.5 g of hazelnuts). In moderate alcohol drinkers, continuous use of small amounts of red wine was allowed. Consumption of commercial bakery (<3 servings/week), soda drinks (<1 drink/day), and spread fats (<1 serving/day) were discouraged. Individuals at high risk of CVD who improved their diet toward a TMD pattern had better markers of heart failure if compared to those following a low fat diet [15]. 

As recently reported by Fernandez-Castillejo et al., MD promotes extra virgin olive oil (EVOO) use. EVOO is rich in monounsaturated fatty acids (MUFA) and poor in saturated fatty acid (SFA). MD also provides several micronutrients like phytosterols, carotenoids, tocopherols and hydrophilic phenols, that have multiple positive health effects in lipid metabolisms [16]. In addition, an adequate daily dietary fiber intake reduces intestinal cholesterol absorption and bile acids reabsorption, with a decrease in cholesterol synthesis and further reduction of LDL cholesterol levels [17].

The association between MD pattern and different type of diabetes has been widely studied. Type 1 diabetes mellitus (DMT1) is a known CVD risk factors and it increases the risk of cardiovascular morbidity and mortality, especially in case of poor glycemic control. MD can improve glycemic control and cardiovascular health in subjects with juvenile DMT1. The SEARCH Nutrition Ancillary Study evaluated the association between nutritional factors and development of cardiovascular risk factors and impaired insulin release in young subjects with DMT1. Study participants were subjects aged less than 20 years at the time of DMT1 diagnosis. Although the number of young people with DMT1 who adopted a MD was low, they displayed lower HbA1c levels and better lipid profile [18].

In an Italian study on subjects with type II diabetes mellitus (DMT2), recruiting 2568 from 57 diabetes clinics, the adherence to MD was calculated with the relative MD score (rMED). Patients who had a higher rMED score followed a less atherogenic and less pro-inflammatory diet, with low calorie and saturated fat intake, high amounts of vegetables, fish, and fiber. This dietary pattern had positive effects on reducing BMI and HbA1c levels, meantime improving lipid profile. This study also emphasized the importance of increasing fiber and reducing saturated fat intake, as they were reported to be respectively lower and higher than recommended in subjects with high rMED scores [19].

Both life style and nutritional habits modifications, and drug therapy can reduce myocardial infarction risk [1,2,4,20]. In 2020, Zurbau et al. conducted a cohort study on 4,031,896 individuals with 125,112 cardiovascular events to evaluate the relationship between incidence and mortality from CVD and fruit and vegetable intake. This study showed a lower risk of CVD, coronary heart disease and stroke in subjects who had higher fruit and vegetables weekly consume. In particular, fruits intake was associated with greater risk reduction. No sources of fruits and vegetables, not even fruit and vegetable juices, were associated with an increased risk for cardiovascular disease. In addition, certain food sources, such as citrus fruits, apples, cruciferous vegetables and green leafy vegetables should be particularly recommended as they may have an even greater effect on preventing CVD risk [21].

La Torre et al. conducted a multicentric case-control study evaluating the association between MD and myocardial infarction: 300 subjects were enrolled in this study, 127 allocated to follow MD, and 173 to low-fat diet. Adherence to MD was related to a reduction in myocardial infarction [22].

In the PREDIMED study, a multicentric, randomized, controlled, parallel-group clinical trial conducted in Spain from October 2003 to June 2009, 930 subjects at high cardiovascular risk (420 men and 510 women) were enrolled. The aim of the study was to test the efficacy of MD on CVD primary prevention [15]. This study showed that subjects at high CVD risk who improved their diet following MD model had lower pro-brain *N*-terminal natriuretic peptide plasma levels if compared to those assigned to a low-fat diet [15]. Brain natriuretic peptide and the *N*-terminal fragment of the prohormone BNP (NT-proBNP) are released predominantly from the ventricular myocardium in response to increased wall stress. NT-pro-BNP has recently been shown to be a negative prognostic factor for mortality and early major cardiovascular events. For the elderly with NT-pro-BNP values above 80 pct, the absolute mortality risk increases to 24.5 percent [23].

The clinical nutrition unit of Careggi Hospital in Florence conducted an open randomized crossover clinical trial called the CARDIVEG study [24]. The study included healthy subjects (18–75 years age) with low-to-moderate cardiovascular risk profile. Participants were overweight, not on drug therapy and had at least one additional metabolic risk factor (impaired fasting blood glucose, high triglyceride levels, elevated total cholesterol, elevated LDL cholesterol, abdominal obesity). Participants were randomly assigned to receive either a calorie-restricted MD or a calorie-restricted lacto-ovo-vegetarian diet. After a two-week run-in phase, the first phase of the dietary intervention started, and after 3 months of dietary intervention, patients switched to the other nutritional regimen for three further months. A total of 118 subjects were enrolled (females 78%, mean age 51.1 years) with a participation rate of 84.7% at the end of the study. The two dietary models had similar effect on body weight reduction (MD: −1.77 kg, vegetarian diet: −1.88 kg), body mass index and fat mass reduction. However, patients following the lacto-ovo-vegetarian diet had a lower low-density lipoprotein cholesterol level, while patients following MD had lower triglyceride levels [24].

In 2019, Pagliai et al. evaluated the effect of 3-month low-calories MD on gut microbiome and short-chain fatty acid production, compared with vegetarian diet. As short-chain fatty acids have an anti-inflammatory effect, their production can be a good marker of anti-inflammatory status [25]. Twenty-three clinically healthy Caucasian subjects (16 women, 7 men; mean age 58.6 ± 9.8 years) were enrolled. As for gut microbiome, no statistically significant differences was evident in the two groups, even if the short duration of the diet can be a possible bias. However, some effect was noted at genus level: subjects following MD had increased gut colonization of *Enterorhabdus*, *Lachnoclostridium*, whereas subjects following vegetarian diet had a prevalence of *Parabacteroides anaerostipes*, *Streptococcus* sp., *Clostridium sensu stricto* and *Odoribacter.* Opposite and statistically significant effect was observed on propionic acid (short-chain fatty acid) production: subjects following MD had a 10% increase in this acid, while those eating a vegetarian diet had a 18% reduction: this finding may further support the anti-inflammatory effect of MD [25].

### 2.2. Mediterranean Diet and CVD Prevention in Pediatric Population

Interest on the healthy-heart effects of MD in pediatric patients is increasingly growing, yet evidence is not as strong as that in adult subjects. Velàzquez-Lòpez et al. evaluated the effect of MD on a sample of 49 children with BMI > 95th percentile and at least one of the criteria for metabolic syndrome [26]. Subjects were randomly allocated to receive standard MD or MD enriched in flavonoids, polyunsaturated fatty acids, fiber and antioxidants. Anthropometric data and body composition, as well as fasting blood samples for glucose, triglycerides, total cholesterol, LDL-cholesterol (LDL-C) and HDL-cholesterol (HDL-C) concentrations were evaluated at baseline and 6 weeks later. The study showed that patients following MD had a higher intake of micronutrients such as zinc, selenium, vitamin E, omega-3 fatty acids and a lower intake of saturated fatty acids; these subjects had better body composition and lower body mass index (BMI) after a 16 weeks intervention [26].

An Italian study analyzed the effect of MD in 6 to 15 years old children with metabolic syndrome by means of a modified version of the KIDMED questionnaire. The study was aimed at assessing adherence to MD of children who did not have access to fast-food restaurants and did not eat cereals and nuts. The study confirmed how increasing adherence to MD reduces incidence of metabolic syndrome, and how the increase of physical activity is associated with much higher adherence to MD [27].

Della Corte et al. enrolled 243 obese patients referring to the hepato-metabolic department of Bambino Gesù Children’s Hospital in Rome from March 2014 to April 2015, to evaluate the effects of MD on non-alcoholic fatty liver disease (NAFLD) [28]. NAFLD can be considered one the most frequent cause of chronic hepatopathy in pediatric age [29]. NAFLD can be defined as an excessive lipid accumulation in the liver in subjects with no co-existing liver disease and without reported excessive alcohol intake (less than 30 g per day in men and less than 20 g per day in women) [30]. This definition has been recently updated for adult subjects, switching from the definition of NAFLD to that of metabolic dysfunction-associated fatty liver disease (MAFLD) [31]. This definition has been recently proposed also for children and adolescents, even if some points have yet to be clarified. The study examined the effects of MD on metabolism and liver damage, also including data on liver biopsies in a relevant number of pediatric patients with NAFLD [32]. All patients underwent blood tests and abdominal ultrasound evaluation. In addition, 100 participants had a liver biopsy performed. Indeed, this was the first study providing histological, instrumental and laboratory evaluation of liver damage in pediatric patients following a MD. The KIDMED questionnaire was used to evaluate adherence to the diet [29]. Data from the study supported the role of MD as a valid tool to prevent liver disease progression in pediatric subjects with NAFLD, as patients with non-alcoholic steatohepatitis had low KIDMED scores. Moreover, patients with poor adherence to MD were at higher risk of liver damage (NAFLD activity score > 5 and grade 2 fibrosis) [27].

A case-control study, conducted on 3- to 5-year-old children belonging to lower-middle socioeconomic class in Spain, with a 3 years follow-up, was designed to evaluate the effect of school gardening on children’s eating habits [33]. Children who regularly participated in school garden activities had higher plant protein and lower animal protein and milk intake than those receiving only school education on healthy eating habits. Though vegetable intake was low in both groups, body mass index was significantly reduced in the intervention group, with lower incidence of overweight and obesity when compared to the control group.

## 3. Plant-Based Diets

Plant-based diets (PBD) have generated great interest because of their potential health effects and, although official estimates are missing, several sources suggest that people are increasingly adopting meat-free diets in industrialized countries [34]. In Germany, approximately 2.5–10% of adult subjects are vegetarians and 0.3–1.6% are vegans [35]. Recent data reported that in the USA, approximately 6% of adults follow a vegetarian diet, with half of them being vegans, and approximately 3% and 2% of 8- to 17-year-old children follow a non-vegan vegetarian and vegan diet, respectively [36]. There are different kinds of PBD, but they are all characterized by an increased intake of plant foods and an avoidance or lower consumption of animal foods [37]. Vegetarian diets are a subset of plant-based diet that, according to Ferdowsian et al., can be divided into four main variants: vegan diet (in which no animal products are allowed), ovo-lacto-vegetarian diet (in which eggs and dairy products are allowed), primary plant diet (similar to the ovo-lacto-vegetarian with small amounts of lean meat) and combination (vegetarian or vegan diet with nuts, soy and fiber) [38]. However, all these diets are based on high daily intake of fruits and vegetables [39,40,41,42], dietary fiber [43], pulses [44], nuts [45] and food rich in unsaturated fatty acids [46]. These plant foods are rich in phytonutrients, minerals, vitamins and fiber, and they have a low energy-density. These features have been favorably associated with lower plasma total cholesterol [47], better glycemic control [48,49], lower blood pressure [50,51], lower incidence of overweight and obesity [52]. PBD increasing popularity seems to be due to the growing concern about planetary sustainability, animal welfare and supposed positive health [53,54]. In fact, in the adult population PBD have been related to a reduced risk of diabetes, obesity and other metabolic disorders [48,55,56]. Furthermore, there is growing evidence that PBD are beneficial in terms of CVD risk reduction [57,58,59,60,61]. 

### 3.1. Plant-Based Diets and CVD Prevention in Adult Population

Several studies report that vegetarian dietary patterns are associated with an improvement in cardio-metabolic risk factors in adult subjects [47,50,62,63]. The same cannot be said for vegan diets: in a Cochrane meta-analysis conducted in 2021, the authors stated that no conclusions can currently be drawn on the effects of vegan dietary interventions on CVD risk factors [64,65]. 

### 3.2. Plant-Based Diets and CVD Prevention in Pediatric Population

In a cross-sectional study, healthy Polish children of white European ethnicity, aged between 5 and 10 years old, were allocated to receive vegetarian, vegan or omnivorous diet for one year [34]. Anthropometric and laboratory test were evaluated at baseline and after 12 months. Vegetarian children showed lower gluteo-femoral adiposity, but similar lean mass and total fat mass. Vegans, on the other hand, showed significantly lower fat indexes in all regions but comparable lean mass. This difference was reduced in the vegetarian group considering the body size, but not in the vegan group (lumbar spine: −5.6%; 95% CI: −10.6, −0.5; total body minus the head: −3.7%; 95% CI: −7.0, −0.4). Vegetarians had higher plasma VLDL, triglycerides and glucose, but lower HDL-C, totacholesterol, serum 25-hydroxyvitamin D and B-12 vitamin. Vegan group were shorter and had higher mean corpuscular volume and homocysteine, but lower plasma HDL-C (−12.2 mg/dL; 95% CI: −17.3, −7.1) and LDL-C (−24 mg/dL; 95% CI: −35.2, −12.9, lower serum B-12 vitamin (−217.6 pmol/L; 95% CI: −305.7, −129.5), 25-hydroxy vitamin D and ferritin, and more prevalent iron deficiency anemia. Both vegetarian and vegan children had lower bone mineral content (BMC) [34]. In line with studies conducted on adult subjects [66], in which higher intakes of non-heme iron (less bioavailable than heme iron) were associated with worse iron status in vegetarians and vegans, this finding was confirmed in this study, suggesting the need for adequate supplementation [34]. Indeed, the German Society for Nutrition had previously stated that it is “difficult or impossible to achieve an adequate supply of some nutrients with a purely plant-based diet”; more in details, vitamin B12 appears to be the most critical nutrient in this category of patients, but so does vitamin D and many others [65]. 

The Vegetarian and Vegan Children Study (VeChi Diet Study) was a larger cross-sectional study which attempted to analyze the effects of vegetarian and vegan diets compared to omnivorous diets in the German pediatric population. A preliminary study involved 139 vegetarian (VG), 127 vegan (VN) and 164 omnivore (OM) children, aged between 1 and 3 years, without any diagnosed diseases that could affect the evaluated parameters. These three groups were compared in terms of energy and macronutrient intake and of anthropometrics data. The final data showed significant differences in macronutrient intake, but not in energy intake or density and anthropometrics between these three groups. OM children had the highest adjusted median intakes of fat (OM: 36.0, VG: 33.5, VN: 31.2% E, *p* < 0.0001), protein (OM: 2.7, VG: 2.3, VN: 2.4 g/kg BW, *p* < 0.0001) and added sugars (OM: 5.3, VG: 4.5, VN: 3.8% E, *p* = 0.002), whereas VN children had the highest adjusted intakes of fiber (OM: 12.2, VG: 16.5, VN: 21.8 g/1000 kcal, *p* < 0.0001) and carbohydrates (OM: 50.1, VG: 54.1, VN: 56.2% E, *p* < 0.0001). In all groups, an average protein intake of 2.3–2.5 times higher than the German reference value was found (1 g protein/kg body weight and day). This finding should be taken into adequate concern, as protein intake excess in the first years of life is related to an increased risk of developing overweight later in life [35].

Alexy et al. analyzed 149 VG, 115 VN and 137 OM children and adolescents, aged between 6 and 18 years. Median protein intake exceeded the German reference value in all groups, being highest in OM and lowest among VN (*p* < 0.02). Carbohydrates intake was higher among VN and VG than among OM (*p* = 0.0002, respectively). On average, fat intake was lower and of better quality among vegan participants, given the higher intake of PUFA and the lower intake of SFA. Total energy intake did not significantly differ among groups. VN showed the lowest LDL-C and non-HDL-C plasma concentrations compared with VG (*p* = 0.0053 and *p* = 0.0041) and OM (*p* = 0.0010 and *p* = 0.0010) children. No significant differences were found in plasma HDL-C and triglycerides levels between the three dietary groups [67].

A recent Finnish cross-sectional study recruited 40 children aged less than 4 years (median age 3.5 years) who had followed an OM (*n* = 24), VG (*n* = 10) and VN (*n* = 6) diet from birth. VN children had significantly lower plasma HDL-C, LDL-C and total cholesterol levels than those of the OM group. Serum vitamin B12 concentrations were adequate in all groups although vegans showed more red blood cell folate than omnivores. No significant differences were found between groups in z-scores of BMI, upper mid-arm circumference, or height [68].

Macknin et al. conducted a 4-week prospective randomized trial to evaluate the effect of a low-fat vegan diet on CVD risk factors in American children, aged 9 to 18 years, with either hypercholesterolemia or weight excess [69]. Overall, 16 participants were allocated to follow a low-fat vegan diet, and 14 to follow the American Heart Association (AHA) advised healthy diet. Children following vegan diet had a statistically significant reduction of BMI Z-score (−0.14), mid-arm circumference (−2.02 cm), weight (−3.05 kg), total cholesterol (−22.5 mg/dL), low density lipoprotein (−13.14 mg/dL), systolic blood pressure (−6.43 mm Hg), hsCRP (−2.09 mg/L), insulin (−5.42 μU/mL), and myeloperoxidase (MPO) (−75.34 pmol/L). However, similar effects were obtained in the AHA diet group, showing significant reduction (*p* < 0.05) in MPO (−69.23 pmol/L), midarm circumference (−1.55 cm), weight (−1.14 kg) and waist circumference (−2.96) [69].

In 2021, Macknin et al. conducted a 52-week prospective randomized trial to evaluate changes in cardiovascular disease risk markers associated with PBD, MD and AHA diet, in 96 patients with obesity and hypercholesterolemia, aged 9–18 years [70]. Each group consisted of 32 patients, who have been evaluated at baseline and after 4 and 52 weeks. PBD consisted of a vegan diet with no added fat and limited salt. Daily vitamin B12 and D were supplemented. The AHA diet was similar to PBD, but some low-fat dairy, non-whole foods, lean meat, selected plant oils and fish were allowed in moderate amounts. MD was similar to the latter, but with higher EVOO, nuts and fish supply. Data collected at 4 weeks revealed significant improvements in (MPO), body weight, and systolic and diastolic blood pressure in all groups. Significant reductions in LDL-C and total cholesterol plasma levels were also observed in PBD and AHA diet groups. The final results showed similar, statistically significant (*p* < 0.05 to <0.001) improvements in all groups: MPO, LDL-C and total cholesterol plasma levels, weight, systolic and diastolic blood pressure were all reduced. Stabilization of BMI-for-age among participants was also observed, as median values modifications were not significant throughout the follow-up period (PBD +1%, *p* = 0.64; AHA −1%, *p* = 0.46; MD −2%, *p* = 0.09) [70].

As shown in Table 1, PBD seem to guarantee a substantial modification or reduction of CVD risk markers. However, the heterogeneity of the studies in the literature, coupled with the often small numbers of participants, represent a bias towards the evaluation of safety and heart-health effects of these diets in the pediatric age. Because of this lack of data, scientific societies have expressed differing positions on PBD. The German Nutrition Society (Deutsche Gesellschaft für Ernährung, DGE) does not recommend a vegan diet for the pediatric population [65], and so does the European Society of Pediatric Gastroenterology, Hepatology and Nutrition (ESPGHAN) for the first two years of life [71]. The American Academy of Nutrition and Dietetics (AAND), on the other hand, has endorsed vegan and vegetarian diets, provided if they are well planned and strictly followed by an expert Pediatrician, during all life stages [72]. Indeed, these diets are often started without precise planning. Micro- and macronutrients excess and/or deficiency, as well as their effects on health, should be foreseen and avoided also using adequate supplementation or fortified foods. In this sense, further and more robust data from longitudinal studies are needed to better assess the effects of PBD on health in all life stages, due to the increasing number of people who decide to follow them.

## 4. DASH Diet

The Dietary Approaches to Stop Hypertension (DASH) diet is a dietary pattern originally developed to achieve better blood pressure (BP) control. [73]. DASH diet encourages daily consumption of fruits, vegetables, and low-fat dairy products. Whole grains, poultry, fish, and nuts intake is promoted, whereas meat, sweets, sugar-containing beverages, total fat, saturated fat and cholesterol intake is reduced [74]. Also, the DASH dietary pattern promotes a higher intake of cardio- protective nutrients such as calcium, magnesium, fiber and vegetable proteins. The mechanism of action through which the DASH diet affects metabolic health and reduces BP is still partly unknown, but several hypotheses have been suggested. Indeed, the high intake of minerals (K^+^, Ca^2+^, Mg^2+^, P) and fiber is likely to exert major beneficial effects on metabolic profiles [75,76]. 

Low Na^+^ and processed food intake, associated with high Ca^2+^ [77], vitamin C [78] and folate [79] intake may have BP lowering effect. Folate is also essential to prevent hyperhomocysteinemia, which is a CVD risk factor. Finally, high intake of pulses and dairy products and low intake of food rich in saturated fats may also account for some metabolic beneficial effects of the DASH diet. Overall, healthy-heart effects of the DASH diet are likely due to a combination of these factors and not just to a single one [80]. 

### 4.1. DASH Diet and CVD Prevention in Adult Population

The AHA recommends DASH diet as a fundamental tool for the non-pharmacological management of blood hypertension, as it is effective in reducing systolic and diastolic BP in hypertensive adult subjects [81]. Many studies have related the DASH diet with other beneficial effects, such as improvement in insulin sensitivity, inflammation [82], oxidative stress [83], glucose and total cholesterol plasma levels [84]. 

A systematic review has shown significant improvement in systolic and diastolic BP, especially in participants with higher BP or BMI at the beginning of the study, and a reduction of total cholesterol and LDL-C plasma levels among adults who followed DASH diet [85]. However, some authors showed no effect of the DASH diet on glucose, triglycerides and HDL-C plasma levels [85]. The DASH diet is associated with increased HDL-C plasma levels [86], a reduction in waist circumference and BMI [87,88] and lower TG, total cholesterol and LDL-C plasma levels [86]. Guo et al. have analyzed the effect of a modified DASH diet, i.e., a diet based on the original DASH diet but with a higher flexibility in food content composition, designed to improve diet feasibility and adherence [73]. Modified DASH diet was significantly associated with reduction in both systolic (SBP) and diastolic blood pressure (DBP; SBP: 3.26 mmHg; DBP: 2.07 mmHg), but not with a reduction in waist circumference [73]. Although participants in these studies had different basal BP value and BMI, and different samples size, we may derive that nutritional management can have a more relevant impact in people with worst initial level of BP or BMI.

### 4.2. DASH Diet and CVD Prevention in Pediatric Population

So far, there are still few studies analyzing the effects of DASH diet in pediatric patients. Nevertheless, according to some studies currently available, DASH diet, due to its high dietary fiber, folate, vitamin C, carotenoids, phytosterols, phytochemicals and anti-oxidants supply [89], may be suitable for children and adolescents with as obesity or insulin resistance and may help maintaining normal growth in these categories of patients. Couch et al. examined the efficacy of a 3-month nutritional intervention based on the DASH Diet versus routine nutritional advice on blood pressure in a cohort of 57 adolescents with hypertension: subjects on DASH diet had higher decrease in SBP z score from baseline to post-treatment than those on traditional nutritional advice, and they also had a greater improvement in diet quality [90]. 

Conflicting data are reported with regard to BP. Saneei et al. demonstrated that DASH diet could prevent the rise in diastolic BP (DBP) but not in systolic BP (SBP); also, no significant changes in glucose and lipid profiles were evident [80]. Mahdavi et al. analyzed the effect of DASH Diet in a group of adolescents with hemophilia, which is an important CVD risk factor [91]. In the intervention group, they found lower SBP, but no effect on DBP; fasting glucose level was improved in the intervention group as well [91]. These different results may be explained by the different adherence to the dietary interventions (lower in the first study, higher in the second one) and the duration of the trials (6 weeks in first study, 10 weeks in the second one).

## 5. Nordic Diet

In the past decades, the Nordic diet has become a healthy regional food option in Northern Europe, where it had been conceived by a group of scientists in the early 2000s [92]. Nordic dietary patterns have been declined in different ways: the Nordic diet (ND) [93], New Nordic diet (NND) [92], Baltic Sea diet [92] and Healthy Nordic diet [94]. Nordic dietary patterns include foods that are consistent with the Nordic dietary guidelines and that are typically taken as part of traditional Nordic eating habits [92]. The Nordic diet shares similar features with the DASH diet [95], but food availability and food culture of the Nordic countries are much more emphasized. The recommended food items include fish/shellfish, rapeseed oil/canola oil (as primary fat sources), low-fat dairy foods, nuts, legumes, berries, fruits (especially pears and apples), vegetables (especially cruciferous and root) and whole-grain cereals (especially barley, rye and oats) [92,94,96]. 

### 5.1. Nordic Diet and CVD Prevention in Adult Population

Many of the recommended food items in Nordic dietary (ND) patterns have been individually associated with a lower risk of CVD, including whole grains, fish, and phytochemicals in vegetables and fruits [97]. A higher daily intake of vegetables and fish and a reduced intake of saturated fat were associated with reduced risk of CVD among adults [39,40,41,42,97,98]. 

The effect of healthy-heart diet based on Nordic Countries habits on decreasing CVD related mortality has been proved decades ago in adult subjects, even before ND had been conceived; back in 1972, in the Northern Karelia Project, an intensive preventive program launched in Finland to lower CVD burden, cholesterol-lowering dietary changes proved to be an effective public health strategy [99]. 

The benefits of ND in the adult population with obesity [100] and diabetes [101,102,103,104] have been recognized in major clinical practice guidelines. In a recent meta-analysis including 15 prospective cohort studies (*n* = 1,057,176 with 41,708 cardiovascular events and 13,121 cases of diabetes) and 6 RCTs (*n* = 717, adherence to Nordic dietary patterns has been associated with overall small important reductions in major CVD outcomes and diabetes incidence [105]. As mentioned earlier, these data refer to the adult population and only a few studies have been published in the literature on the effects of the ND in the pediatric population.

### 5.2. Nordic Diet and CVD Prevention in Pediatric Population

Historically, the effects of healthy-heart diet based on Nordic Countries habits in CVD prevention in pediatric patients were evaluated in the Special Turku Coronary Risk Factor Intervention Project (STRIP), a randomized controlled trial involving 1116 children recruited at 5 months of age and randomly assigned to receive either a heart-healthy diet characterized by low intake of saturated fat and cholesterol and dietary counselling or only basic health education (control group). The STRIP study demonstrated that low-saturated fat, low-cholesterol diet counselling started in early childhood has a significant lowering effect on serum cholesterol levels, and it exerts positive effects on endothelial function. These positive effects are maintained for many years [106]. Moreover, it does not interfere with children’s growth and cognitive, neurological or pubertal development [107]. After a 26-years follow up, previously observed intervention effects during the 20-years nutritional counselling were maintained despite 6 years of intervention withdrawal, thus confirming that nutritional intervention and counselling started early in childhood prove to be effective in CVD risk prevention throughout adulthood [108]. In 2014 Andersen et al. conducted a cluster-randomized, controlled, unblinded, cross-over design study (Optimal Well-Being, Development and Health for Danish Children through a Healthy New Nordic Diet (OPUS) School Meal Study) with the aim to investigate the impact on nutrient and food intake by introducing school meals based on ND principles, covering all snacks and lunch during the school day, in 46 school classes from 9 different Danish schools over two 3-month periods. 834 children aged 8–11 years randomly received either their usual packed lunch brought from home (control) or the ND school meal (intervention group) in the intervention group had higher intakes of fish, potatoes, vegetables, cheese (and dairy products), eggs and beverages (excluding milk) and lower intakes of fats (less saturated fat), bread and other cereal products than those in the control group (*p*: <0.05). No differences were found in the intake of fruit, milk, meat, poultry, and in the average daily energy intake. Furthermore, fat and protein energy distribution were significantly different (*p* = 0.0001), with children who reported a higher average energy intake from protein and a lower energy intake from fat while on ND. Regarding micronutrient intake, major differences were observed for iodine and vitamin D (both *p* < 0.0001), due to the higher fish consumption by ND. Although some changes found in the study are modest and partially influenced by the food consumed outside school, the authors concluded that adherence to ND, even for a part of the daily meals, can bring heart-healthy benefits [109]. 

In 2015, Sørensen et al. analyzed data from 726 children aged 8–11 years in a 3-month intervention trial [24]. The main aim of the study was to investigate whether ND intake at school influenced *n*-3 LCPUFA and iron status, and if these nutrients variations could correlate with cognitive performance changes. Both baseline EPA + DHA status and the intervention-induced increase in EPA + DHA status were positively associated with school performance. Reported fish intake was positively correlated with DHA and EPA status (*p* < 0.001). Furthermore, those who had consumed fish had higher DHA and EPA status (*p*: <0.001) and lower *n*-6:*n*-3 PUFA ratio (*p*: <0.001) than children who did not eat fish [106]. LCPUFAs are related to a reduction in CVD risk factors both in adult and pediatric patients, and even if the study did not analyze this correlation, it laid the foundations for possible further research [110].

Agnihotri et al. recently analyzed children’s adherence to ND at 6 months, 18 months, 3 years and 7 years, and its potential association with childhood overweight at 8 years, using data from 14,989 patients collected by a large cohort study (Norwegian Mother, Father and Child Cohort Study) [111]. In unadjusted analyses, adherence to ND at 6 months showed an inverse correlation with the risk of overweight at 8 years, when comparing high versus low ND adherence (odds ratio = 0.81, 95% CI [0.70, 0.94]), and in the continuous score (odds ratio = 0.95, 95% CI [0.91, 0.98]). However, children ND adherence up to 7 years of age was not associated with the risk of overweight at 8 years, after adjusting for potential confounders. The results observed in the unadjusted models were mostly explained by the mother’s level of education and the mother’s pre-pregnancy weight status. In fact, the authors found that mothers of these children were less commonly overweight/obese, had higher education and had a healthier diet during pregnancy [112]. 

ND has been also evaluated with regard to blood pressure in epidemiological studies [109] and in clinical trials [113]. Based on the assumption that low-birth weight is a known risk factors for developing blood hypertension in the following years [114,115], Meinilä J et al. tried to assess whether birth weight modifies the correlation between ND and blood pressure [116]. Finnish women and men (*n* = 960) born in 1934–1944 were administered questionnaires and underwent clinical visits in 2001–2004 and 2011–2013. Linear regression was used to investigate the interactions between ND (measured by the Baltic Sea diet score (BSDS)) and birth weight on blood pressure modifications over a 10-year follow-up. Birth weight and ND exhibited a significant interaction on SBP (*p* = 0.02) and pulse pressure (PP) (*p* < 0.01), but not on DBP and mean arterial pressure (MAP). Based on birth weight, the participants were divided into three groups. In the lowest birth weight group (men < 3061 g, women < 2951 g), predicted SBP decreased across Baltic Sea Diet Score (BSDS) (lowest (T1): 155 mmHg, highest (T3): 145 mmHg, *p* = 0.01), as well as the predicted PP (T1: 71 mmHg, T3: 63 mmHg, *p* < 0.01). In the middle birth weight group, predicted SBP increased across BSDS thirds (T1: 151 mmHg, T3: 155 mmHg, *p* = 0.02) as did predicted PP (T1: 67 mmHg, T3: 71 mmHg, *p* < 0.01). In the highest birth weight group (men > 3959 g, women > 3810 g), no associations were found. According to these results, the authors concluded that benefits on SBP and PP can be achieved through a healthy diet and the effects are greater among individuals with low birth weight [116]. 

In a recent cross-sectional study by Rodríguez-Borjabad et al., dietary intake and lipid profiles of 4–18-year-old children from Norway and Spain who ND and MD respectively, with (*n* = 114) and without (*n* = 145) Familial hypercholesterolemia (FH), were assessed [117]. None of the participants had received lipid-lowering drugs prior to the study visit. The results showed that the Spanish FH cohort had higher dietary intake of total fat (mostly monounsaturated fatty acids (MUFA)), cholesterol and fiber, but a lower dietary intake of polyunsaturated fatty acids (PUFA) compared to the Norwegian FH cohort. No significant differences were found in the percentage of daily energy provided by proteins and carbohydrates. In the Spanish cohort, daily energy percent of fats and MUFAs were negatively associated with LDL-C and apolipoprotein B and positively associated with apolipoprotein A1 and HDL-C. In the Norwegian cohort, daily energy percentage from fats, SFAs, PUFAs and MUFAs were positively associated with apolipoprotein B, total cholesterol and LDL-C. Indeed, overall correlations were weak. Stepwise linear regression analysis revealed no effect of diet composition on LDL-C and total cholesterol levels when the Spanish and Norwegian FH cohorts were examined independently, but when both were pooled, a weak inverse effect of carbohydrates and proteins intake on LDL-C was found (coefficient (*p*-value); −0.06 (0.03), −0.18 (0.001) respectively), suggesting a direct effect of fats. In this sense, the authors concluded that an impact of diet on lipid alterations in children with FH cannot be excluded, even though these are due to a genetic defect. Finally, despite small children sample size, limited data on physical activity, and the use of different questionnaires to collect dietary data, this was one of the first studies aimed at investigating ND and its possible effect on lipid profiles in children with FH [118]. 

The results of the main studies on ND in pediatric patients are summarized in Table 2. Although the literature shows that several plant foods based dietary patterns are associated with lower risk of CVD in adults, the applicability of these patterns in Northern European countries is limited by the availability/cost of specific foods and cultural preferences and choices [119,120]. Therefore, further well-designed prospective studies with the aim to determine the effects of ND on nutritional and metabolic parameters and possible correlations with CV risk factors in the pediatric population of these countries are needed.

## 6. Low-Carb Diet

Hypertension, hyperlipidemia, abdominal obesity, hyperinsulinemia and type 2 diabetes mellitus (T2DM) are among the main CVD factors. Adult subjects who follow a healthy diet, with a very close control of carbohydrate quality and quantity, combined with increased physical activity and healthy lifestyle, have an improvement in insulin resistance, lipid profile and in weight loss [121]. In a systematic review, low carbohydrate diet (LCD) has been defined as a nutritional regimen providing no more than 60 g/day of carbohydrates (CHO) [122]. LCD could be further classified depending on different percentage of carbohydrate restriction: LCD provides 26–45%, very low-carb diet (VLCD) 10–25% daily carbohydrate intake, respectively [118]. This diet can offer many potential advantages, such as lower basal insulin level, higher water loss and increased glycogen store utilization, thus promoting faster and longer lasting satiety, and limiting food variety. The CHO restricted diet provides reduced intake of foods rich in refined CHO, such as added sugars and fructose, by replacing them with CHO sources derived from leafy greens, vegetables, nuts, yogurt and low-glycemic fruits (i.e.,: apples and berries). Saturated fat intake is usually limited to less than 10% of daily energy intake. Proteins and fats account for the highest percentage of daily energy intake: protein sources include meat, fish, eggs and poultry while fat source derives form olive oil, walnut oil, and other sources of poly- and mono-unsaturated fatty acids. Saturated fats intake is not recommended [123]. 

LCD has been proposed as an alternative to conventional diets, as it can positively affect lipid profile [124]. Finally, there is increasing evidence of LCD effectiveness also in pediatric age, with promising future perspectives [125,126].

### 6.1. Low Carbohydrate Diet and CVD Prevention in Adult Population

Many studies carried out in adult subjects show a strong beneficial impact of LCD on major metabolic risk factors, by lowering serum triglycerides, increasing serum high density lipoprotein (HDL) levels, and by stimulating a better insulin post-prandial response [127,128,129,130,131]. Zeybek et al. have demonstrated normalization of some parameters related to ventricular right diastolic function, such as isovolumetric relation time of the right ventricular free wall and lateral tricuspid annulus, which are overall altered by overweight, after six months of LCD in adult subjects with weight excess [130]. Indeed, it is crucial to early detect these cardiac abnormalities, to promptly start adequate weight reduction, thus tackling the progression of heart damage.

In conclusion, LCD is an effective tool in reducing insulin resistance, dyslipidemia and weight excess both in adult and in children [2,3,4], thus playing a key role in CVD risk prevention.

### 6.2. Low Carbohydrate Diet and CVD Prevention in Pediatric Population

Few studies, evaluating the efficacy and safety of LCD in children, tested different amounts and quality of CHO, and different percentage of other nutrients (in particular proteins) [126]. It is well known that macronutrients restriction may have negative effects on children growth and neurodevelopment, thus macronutrients intake should be thoroughly monitored by nutrition skilled pediatricians and never started before two years of age.

LCD is not recommended for weight reduction in adolescents, due to a too restricted daily calorie intake [121]. Demol et al. showed no advantages of LCD on weight loss or on BMI in children with weight excess, but they found a decrease in serum total cholesterol, LDL-C, HDL-C and triglycerides levels [127]. They also found a significant glycemia, insulin and HOMA (an indirect sign of insulin resistance) levels reduction [127].

Unlike these findings, in a randomized study conducted in a cohort of adolescents with obesity, Sondike et al. showed an important decrease of weight and BMI in the obese children group following LCD. In this randomized controlled trial, they compared the effects of LCD to those of a low fat diet on weight loss and serum lipids in obese adolescents. In the intervention group, adolescents’ daily carbohydrate intake was below 20 g for 2 weeks, then less than 40 g of carbohydrates for 2 further weeks, whereas subjects in the control group were advised not to exceed 30% of total daily energy deriving from fat. Subjects in the intervention group lost more weight and had better improved in non-HDL cholesterol plasma levels, whereas only subjects in the control group had an improvement in LDL cholesterol plasma levels [127]. Likewise, Gow et al. also observed a significant beneficial effect on weight loss and BMI. They also hypothesized that, besides diet, also pubertal and glycemic status may affect weight loss [132].

Up to now, long-term safety of LCD has not yet been fully clarified, either because a reduced intake of certain foods might have negative impact on the adolescents’ growth [133] or it might be really hard for adolescents to follow a restricted diet for a long period of time [134].

## 7. Ketogenic Diet

Ketogenic diet (KD) is a low-carbohydrate, high-fat dietary pattern, with adequate protein supply. The “ketogenic ratio”, calculated as grams of fat to grams of protein and CHO, can range from 1:1 to 4:1 [135]. At present, four different types of KD have been described: the classic KD, with a 4:1 ketogenic ratio; the modified Atkins diet (MAD), with a 1.1:1 ketogenic ratio; the medium chain triglyceride diet (MCTD), where 60% of the energy is given by MCTs, and the low glycemic index (GI) regimen, based on food with a low glycemic index [136].

CHO restriction activates gluconeogenesis and ketogenesis. Indeed, in lack of glycogen, fatty acids stores are catabolized to ketone bodies (acetone, acetoacetate and beta-hydroxybutyrate), which are used as the main energy sources. Ketosis state is defined by a serum ketones concentration of at least 0.5–3.00 mmol/L [137].

### 7.1. Ketogenic Diet and CVD Prevention in Adult Population

In adults, KD has been tested in adult patients with obesity (BMI > 30 kg/m^2^), overweight with co-morbidities (hypertension, non-insulin dependent diabetes mellitus, dyslipidemia, obstructive sleep apnea syndrome, metabolic syndrome, severe osteopathy or arthropathy, NAFLD), and in patients eligible for bariatric surgery [138]. Recently, Kathryn Dowis et al. analyzed the potential beneficial effects and impact of KD on gut microbiome, epigenome, diabetes, weight loss and cardiovascular diseases [139]. Several studies, focused on the cardiovascular effects of KD, reported a reduction in abdominal fat and BMI, a decrease of SBP and DBP and modifications of lipid profile, with reduction of total cholesterol, triglycerides, and LDL-C and increase of HDL-C values [140,141,142,143].

### 7.2. Ketogenic Diet and CVD Prevention in Pediatric Populaton

At present, refractory epilepsy is one of the main clinical applications of the ketogenic nutritional protocol in pediatric patients. It was introduced in 1921, as an effective, non-pharmacological treatment in infants with epilepsy non responsive to at least two or more antiepileptic drugs. In fact, in the brain, ketone bodies exert their anti-inflammatory action crossing the blood-brain barrier (BBB) [144,145]. Though KD may have several side effects, with gastrointestinal, hepatic, cardiovascular, renal, dermatological, hematologic and bone involvement, the majority of them can be easily treated, making KD a safe “natural” optional treatment [146].

KD, due to its high-fat content, is associated with hyperlipidemia, strongly connected with CVD. Several studies reported an increase of VLDL, LDL-C, triglycerides, total cholesterol and total apo-B plasma levels and a decrease of HDL-C in children with refractory epilepsy after six-months of KD. [147,148]. However, the size of LDL particles is progressively and constantly reduced during KD treatment in these patients; considering that small LDL are more atherogenic because of their capacity to migrate more easily in the subendothelial layer, this aspect must be taken into account when prescribing KD for a long period of time [149].

On the other hand, as Liu et al. demonstrated in children with pre-existing dyslipidemia, ketogenic diet (with a high unsaturated fat content) may also have some beneficial effects, by improving total cholesterol, low-density lipoprotein, total cholesterol and high-density lipoprotein serum levels. Liu et al. analyzed retrospectively 1660 children treated with KD [150]. KD adopted by children and adolescents with weight excess resulted in BMI reduction, due to significant weight loss, with improvement of the lipid profile (decrease in LDL-C and increase in HDL-C cholesterol plasma levels), reduction of SBP and DBP, and improvement of metabolic parameters such as insulin sensitivity and resistance [151]. In subjects with obesity, adiponectin plasma levels are usually low and proinflammatory cytokines, such as leptin, resistin, IL-6, IL-10 and tumor necrosis factor (TNF-α) are usually elevated, thus producing a pro-inflammatory metabolic pattern [152]. KD have antioxidative and anti-inflammatory proprieties, by reducing reactive oxygen species (ROS) and TNF-α production [153]. In this context, KD can be considered a useful tool to reduce inflammatory state, which is linked to obesity, atherosclerosis, and hypertension.

Vascular function can be altered in epileptic children following a KD. In this regard, Kapetanakis et al. analyzed 26 children with epilepsy on KD and they found that carotid distensibility worsened after 12 months of KD treatment, whereas cIMT (carotid intima-media-thickness) was not significantly altered. After one year they found an increase in LDL cholesterol and apolipoprotein B plasma levels, but these values returned to normal range after two years of treatment [154]. Despite this, Ozdemir R et al. reported no significant changes in cIMT, aortic and carotid elasticity in pediatric patients on KD [155].

Despite the effects on lipid profile and subsequent increase in CVD risk, KD is used in children with refractory epilepsy, as the effects on seizures frequency and types are greater than the increase in CVD risk. However, a close clinical follow-up is advised for pediatric patients on KD to monitor and prevent its multisystemic complications, including CVD effects.

## 8. Paleolithic Diet

Recently, the Paleolithic diet (PD) has been proposed as a promising dietary pattern for its presumed beneficial effects on CVD risk factors. PD is based on the intake of vegetables (tubers, seeds, nuts, wild-grown barley that was pounded into flour, legumes, and flowers), fruits, fish, lean meat, and on the avoidance of grains, dairy products, processed food and added sugars and salt [156].

### 8.1. Paleolithic Diet and CVD Prevention in Adult Population

In a randomized controlled single-blinded pilot study in adult patients with at least two characteristics of the metabolic syndrome, Boer et al. showed that patients following PD had an improvement in several CVD risk factors. After two weeks, lower SBP and DBP, lower LDL-C and total cholesterol, and higher HDL-C plasma levels became evident [157]. In a two-years randomized controlled trial conducted in Sweden on obese post-menopausal women, aimed at evaluating the beneficial effects of PD on the CVD health, patients following PD had a reduction of fat mass, abdominal obesity and triglyceride levels if compared to the control group following ND [158]. PD effects have been also tested in adult subjects with diabetes. In a randomized cross-over study, adult subjects with diabetes on PD had lower HbA1C and triglycerides plasma levels, lower DBP, and improved BMI and waist circumference values after 3 months [155]. Masharani et al. conducted a randomized controlled trial in patients with T2DM following PD reporting a significant improvement in insulin sensitivity [159]. Lindeberg et al. compared the effect of a 12 weeks PD and MD in a cohort of 29 patients with ischemic heart disease and glucose intolerance or type 2 diabetes. In the PD group, significant improvement in glucose tolerance was reported, when compared to MD group [160]. In a systematic review focused on PD nutritional approach in adult subjects with metabolic syndrome, PD was related to an improvement of waist circumference, triglycerides plasma levels and BP (both systolic and diastolic), whereas no statistically significant changes were reported for HDL-C and fasting glucose plasma levels [161].

### 8.2. Paleolithic Diet and CVD Prevention in Pediatric Population

At present, there is no clear evidence of PD healthy effects in the pediatric population. However, it might be helpful to understand how this nutritional protocol could influence also children health and CVD risk factors. Indeed, the favorable consequences of PD in adults are demonstrated by different studies. As a matter of fact, our ancestors’ original diet was lacking in added sugars and salt, processed foods and dairy products which are definitely associated with high risk of CVD, currently considered as the leading cause of mortality worldwide [162].

## 9. High protein Diet

Protein average intake differs between various population groups, according to gender, age, and peculiar life conditions (e.g., adulthood, pregnancy, infancy, childhood) As for adulthood, the adequate daily protein intake should be 0.83 g/kg, regardless of gender [163]. The majority of authors agree that diets providing more 1.5 g protein/kg/day should be considered to be a high-protein diet [164].

High Protein Diet and CVD Prevention in Adult Population

Since the 1960s, high protein dietary models have been used as nutritional regimens in patients with weight excess, as well as to ameliorate T2DM. In a systemic review aimed at evaluating the effects of a high-protein diet (HPD) on glycemic control, insulin resistance and blood pressure in T2DM patients, the authors found a remarkable reduction in LDL-C, total cholesterol, triglycerides plasma levels, and HOMA-IR index reduction (Homeostatic Model Assessment for Insulin Resistance), but no significant result has been recorded with regard glycemic control (fasting blood glucose and HbA1c values), HDL-C plasma levels, and blood pressure. These findings suggest a role of HPD in improving insulin resistance and lipid profile [165]. In an open-label, single-center randomized controlled dietary trial in obese women, a protein enriched diet has proved to be more effective in improving insulin resistance and glycemic values than MD [166].

Also, HPD may play a role in preventing the metabolic syndrome, a major cardiovascular risk factor, which includes central adiposity and almost two of the following four factors: low HDL-C and high triglycerides levels, high blood pressure, and hyperglycemia [167]. In Mexico, where the metabolic syndrome prevalence is above 49.8%, obese people on a high protein diet (1.34 g/kg), showed a significantly greater weight loss than participants who followed a standard protein diet (0.8 g/kg) [168]. So, if on the one hand HPD is widely used by adult people to lose weight and ameliorate glucose plasma levels, on the other hand a high intake of protein foods is not so feasible in children. Indeed, Elke Dorenbos et al. have tried to offer a high protein and low glycemic index diet in overweight or obese adolescents with insulin resistance, but no effects were observed because of the poor dietary compliance [169]. Vajihe Izadi et al. performed a randomized controlled trial with the aim to understand the effects of HPD on improving anthropometric measurements or other CVD risk factors, such as SBP and DBP, weight, waist circumference, LDL-C and HDL-C, total cholesterol, fasting blood glucose (FBG), triglyceride (TG), Apo protein A and B100, fasting blood insulin, Apo A/Apo B, TC/HDL ratio, TG/HDL ratio, non HDL cholesterol and HOMA-IR index [170]. They did not report any significant effect of adherence to an HP diet in improving anthropometric measurements or other CVD risk factors among obese and overweight children [171].

However, a recent meta-analysis, including eight different studies, demonstrated that only a short-term HPD was able to improve BMI in overweight and obese children, without beneficial effects on plasma lipid profile [172]. Finally, further studies are needed to assess whether HPD, combined with healthy lifestyle, can also affect children’s cardiovascular health.

## 10. Conclusions

Nutritional intervention plays a pivotal role in CVD risk prevention and treatment, especially in children and adolescents [1,2,4,5]. In the last years, focus has shifted from single nutrients and single food role to the so called dietary patterns [9]. The AHA recently evaluated the most frequently adopted dietary patterns in the adult population and identified those which have a stronger adherence with the 2021 AHA Dietary Guidance [9], so as to promote cardiometabolic health. In Table 3, we summarize the effect of different dietary patterns on CVD risk in adult and pediatric subjects.

A limitation to the use of dietary patterns may be that geographical environment and specific population can affect cardiovascular risk factors: there are important differences in the prevalence, management and outcomes of atherosclerosis-related diseases across different geographic regions; thus calibrated, contemporaneous country-specific charts for all European countries have been developed [1]. Dietary patterns vary depending on geographic regions as well, and they can be considered a part of CVD risk. For instance, in the Reasons for Geographic and Racial Differences in Stroke (REGARDS), a national, population-based, longitudinal study of white and black adults aged ≥45 years, a typical dietary pattern of the southern United States was associated with higher CVD risk, whereas MD to lower CVD risk [173]. Dietary patterns have been widely adopted also for pediatric subjects [6]. MD, DASH diet, ND and some PBDs seem to be the more promising heart-healthy dietary patterns, even if further studies are needed to better analyze their implications in developmental age. Moreover, most studies conducted in pediatric patients are focused on primary prevention, and they usually have a limited follow up; on the contrary, studies concerning adult subjects can analyze the long term effects of dietary patterns on more specific CVD markers, as well as in a wider patients’ cohort. Children and adolescents are not little adults, therefore adequate growth parameters and neurodevelopmental milestones achievement must always be a priority when dealing with these subjects.

## Figures and Tables

**Table 1:** Plant based studies in pediatric patients.

Type of Study	Population	Results	Author
Cross-sectional study	63 vegetarians, 52 vegans and 72 omnivores after having followed the respective diet for at least 1 year (healthy children) 5–10 years	Compared to the omnivore group, the vegan and vegetarian group was leaner, with lower levels of HDL-, LDL- and Total-cholesterol, but also shorter, with lower BMC and blood levels of vitamin B12 and D.	Desmond MA 2021 [34]
Cross-sectional study	139 vegetarians, 127 vegans and 164 omnivores (healthy children) 1–3 years	Significant differences in macronutrient intake: protein, fat, added sugars (OM > VG > VN). Carbohydrates and fiber (VN > VG > OM). No significant differences in energy intake or density and anthropometrics.	Weder S 2019 [66]
Cross-sectional study	149 vegetarians, 115 vegans and 137 omnivores (healthy children) 6–18 years	Significant differences in macronutrient intake: protein (OM > VN > VG). Carbohydrates (VN > VG > OM). Fat (OM > VN > VG). VG showed the lowest LDL-C and non-HDL-C concentrations compared with VN and OM. No significant differences were found in HDL-C, triglycerides and energy intake.	Alexy U 2021 [67]
Cross-sectional study	Omnivores 24, vegetarians 10, vegans 6 who followed their respective diets from birth <4 years (median age 3.5 years old)	Vegans had significantly lower plasma HDL-C, LDL-C and total cholesterol levels than the omnivore group, with a median total cholesterol level of 2.85 mmol/L.	Hovinen T 2021 [68]
4-week prospective randomized trial	16 vegans, 14 AHA diet hypercholesterolemic and obese children 9–18 years	Children on PB had 9 and children on AHA had 4 statistically significant (*p* < 0.05) beneficial changes from baseline (mean decreases): BMI Z-score^PB^ (−0.14), systolic blood pressure^PB^ (−6.43 mm Hg), total cholesterol^PB^ (−22.5 mg/dL), LDL^PB^ (−13.14 mg/dL), hsCRP^PB^ (−2.09 mg/L), insulin^PB^ (−5.42 μU/mL), myeloperoxidase^PB/AHA^ (−75.34/69.23 pmol/L), mid-arm circumference^PB/AHA^ (−2.02/−1.55 cm), weight^PB/AHA^ (−3.05/ −1.14 kg) and waist circumference^AHA^ (−2.96 cm).	Macknin M 2015 [69]
52-week prospective randomized trial	32 vegans, 32 AHA diet, 32 MED diet, hypercholesterolemic and obese children 9–18 years	Similar statistically significant (*p* < 0.05 to <0.001) improvements were found in all groups in the CVD risk markers: low-density lipoprotein, myeloperoxidase (MPO), total cholesterol, weight, systolic and diastolic blood pressure. The stabilization of BMI-for-age among participants was also observed. No significant changes in hsCRP, HDL and insulin were detected during the study in all study groups	Macknin M 2021 [70]

HDL: high density lipoprotein; LDL: low density lipoprotein; HDL-C: high density lipoprotein -cholesterol; LDL-C: low density lipoprotein -cholesterol; CVD: cardiovascular; OM: omnivore; VN: vegan; VG: vegetarian; AHA: American Heart Association; MED:Mediterranean.

**Table 2:** Nordic diet studies in pediatric patients.

Type of Study	Population	Results	Author
Randomized controlled trial	834 children randomly received their usual packed lunch brought from home (control) or the ND school meal over two 3-month periods 8–11 years	During the ND period, children had higher intakes of fish, vegetable, cheese, potatoes, eggs and beverages (excluding milk) and lower intakes of fats (less saturated fat), bread and other cereal products than in the control period (all, *p* < 0.05).	Andersen 2014 [109]
Randomized controlled trial	726 children randomly received their usual packed lunch brought from home (control) or the ND school meal over a 3-month period 8–11 years	Both baseline EPA + DHA status and the intervention-induced increase in EPA + DHA status was positively associated with school performance. Those who had consumed fish had higher DHA and EPA status (both *p* < 0.001) and lower *n*-6:*n*-3 PUFA ratio (*p* < 0.001).	Sørensen 2015 [110]
Cross-sectional study based on data from a large prospective cohort study	14.989 children <8 years	In final analysis, child ND adherence up to 7 years of age was not associated with odds of overweight at 8 years after adjusting for potential confounders.	Agnihotri 2021 [112]
Cross-sectional study	960 partecipants born in 1934–1944	Benefits on SBP and PP can be achieved through ND and the effects are greater among individuals with low birth weight.	Meinilä 2021 [116]
Cross-sectional study	4–18-year-old children with (*n* = 114) and without (*n* = 145) FH who followed Nordic and Mediterranean pattern diets	An impact of diet on lipid alterations in children with FH cannot be excluded, even though these are due to a genetic defect.	Rodríguez-Borjabad et al. 2021 [117]

FH: familial hypercholesterolemia; ND: nordic diet; EPA: eicosapentaenoic acid; DHA: docosahexaenoic acid.

**Table 3:** Effects of dietary patterns in adult and pediatric subjects.

Dietary Pattern	Main Characteristics	Main Effects in Adult Subjects	Main Effects in Pediatric Subjects
Mediterranean Diet	high daily intake of vegetables, fruits, pulses, and cereals (mainly in raw, unprocessed forms) low intake of meat and meat-derived products low to moderate intake of dairy products. moderate/high fish intake predominant use of unsaturated lipids, especially in the form of olive oil	Positive effects on markers of heart failure, reduction of triglycerides plasma levels. Reduced BMI and HbA1c levels and improved lipid profile in subjects with DMT2 Reduction of myocardial infarction risk	higher intake of micronutrients (zinc, selenium, vitamin E, omega-3 fatty acids) lower intake of saturated fatty acids; better body composition and lower body mass index reduced risk of metabolic syndrome. lower incidence of overweight and obesity
Plant based Diet	vegan diet (no animal products are allowed) ovo-lacto-vegetarian diet (eggs and dairy products are allowed) primary plant diet (similar to the ovo-lacto-vegetarian with small amounts of lean meat) combination (vegetarian or vegan diet with nuts, soy and fiber)	vegetarian dietary patterns are associated with improvement in cardio-metabolic risk factors	better lipid profile Lower systolic blood pressure Lower Body Mass Index
DASH Diet	daily consumption of fruits, vegetables, and low-fat dairy products. Whole grains, poultry, fish, and nuts intake is promoted, reduced intake of meat, sweets, sugar-containing beverages, total fat, saturated fat and higher intake of cardio- protective nutrients such as calcium, magnesium, fiber and vegetable proteins	improvement in insulin sensitivity, inflammation oxidative stress, glucose and total cholesterol plasma levels	may help maintaining normal growth in pediatric patients with obesity and/or insulin resistance
Nordic Diet	include foods that are consistent with the Nordic dietary guidelines The recommended food items include fish/shellfish, rapeseed oil/canola oil (as primary fat sources), low-fat dairy foods, nuts, legumes, berries, fruits (especially pears and apples), vegetables (especially cruciferous and root) and whole-grain cereals (especially barley, rye and oats)	Reduced cardiovascular disease related mortality. Small reduction in diabetes incidence.	An impact of diet on lipid alterations in children with FH cannot be excluded, even though these are due to a genetic defect. Benefits on SBP and PP can be achieved through ND and the effects are greater among individuals with low birth weight. Better LCPUFA profile (higher intake of omega-3)
Low-carb Diet	a nutritional regimen providing no more than 60 g/day of carbohydrates (CHO) Low Carb Diet could be further classified depending on different percentage of carbohydrate re-striction: LCD provides 26–45%, very low-carb diet (VLCD) 10–25% daily carbohydrate intake, respectively reduced intake of foods rich in refined CHO, such as added sugars and fructose, by re-placing them with CHO sources derived from leafy greens, vegetables, nuts, yogurt and low-glycemic fruits	beneficial impact of LCD on major metabolic risk factors, by lowering serum triglycerides, increasing serum high density lipoprotein (HDL) levels, and by stimulating a better insulin post-prandial response. Beneficial effect on lowering BMI	Macronutrients restriction may have negative effects on children growth and neuro-development, thus macronutrients intake should be thoroughly monitored by nutrition skilled pediatricians and never started before two years of age. Up to now, long-term safety of LCD has not yet been fully clarified, a reduced intake of certain foods might have negative impact on the adolescents’ growth or it might be really hard for adolescents to follow a restricted diet for a long period of time
Ketogenic Diet	Low-carbohydrate, high-fat dietary pattern, with adequate protein supply. The “keto-genic ratio”, calculated as grams of fat to grams of protein and CHO, can range from 1:1 to 4:1. Four different types of KD have been described: the classic KD, with a 4:1 ketogenic ratio; the modified Atkins diet (MAD), with a 1,1:1 ketogenic ratio; the medium chain triglyceride diet (MCTD), where 60% of the energy is given by MCTs, and the low glycemic index (GI) regimen, based on food with a low glycemic index	reduction in abdominal fat and BMI decrease of SBP and DBP modifications of lipid profile, with reduction of total cholesterol, triglycerides, and LDL-C and increase of HDL-C values	At present, refractory epilepsy is one of the main clinical applications of the keto-genic nutritional protocol in pediatric patients. Despite the effects on lipid profile and subsequent increase in CVD risk, KD is used in children with refractory epilepsy.
Paleolithic Diet	based on the intake of vegetables (tubers, seeds, nuts, wild-grown barley that was pounded into flour, legumes, and flowers), fruits, fish, lean meat. avoidance of grains, dairy products, processed food and added sugars and salt.	improvement in several CVD risk factors in subjects with at least two characteristics of the metabolic syndrome.	No clear effect
High Protein Diet	diets providing more 1.5 g protein/kg/day should be considered to be a high-protein diet.	reduction in LDL-C, total cholesterol, triglycerides plasma levels, and HOMA-IR index.	improved BMI in overweight and obese children, without beneficial effects on plasma lipid profile

CVD: cardiovascular; KD: ketogenic diet; BMI: body mass index; DMT2: type II diabetes mellitus; LCPUFA: long chain polyunsaturated fatty acids; FH: familial hypercholesterolemia; ND: nordic diet; LDL-C: low density lipoprotein -cholesterol; MCT: medium chain triglyceride; HOMA-IR: homeostatic model assessment for insulin resistance.

## Data Availability

Not applicable.

## Abbreviations

AAND	American Academy of Nutrition and Dietetics
AHA	American Heart Association
ApoB	Apolipoprotein B
BBB	Blood Brain Barrier
BMI	Body Mass Index
BP	Blood Pressure
BNP	Brain Natriuretic Peptide
BSDS	Baltic Sea Diet Score
CHO	Carbohydrate
CVD	Cardiovascular Disease
DASH	Dietary Approaches to Stop Hypertension
DBP	Diastolic Blood Pressure
DHA	Docosahexaenoic Acid
DMT1	Diabetes Mellitus Type 1
DMT2	Diabetes Mellitus Type 2
EPA	Eicosapentaenoic Acid
EVOO	Extra Virgin Olive Oil
GI	Glycemic Index
HDL	High Density Lipoprotein
HOMA-IR	Homeostasis Model Assessment Insulin Resistance
KD	Ketogenic Diet
LCD	Low Carbohydrate Diet
LCPUFA	Long Chain Polyunsaturated Fatty Acids
LDL	Low Density Lipoprotein
MAD	Modified Atkins Diet
MAFLD	Metabolic dysfunction-Associated Fatty Liver Disease
MAP	Mean Arterial Pressure
MCTD	Medium Chain Triglycerides Diet
MED	Mediterranean
MPO	Myeloperoxidase
MUFA	Monounsaturated Fatty Acids
red	Relative Mediterranean Diet Score
NAFLD	Non-Alcoholic Fatty Liver Disease
ND	Nordic Diet
NND	New Nordic Diet
PBD	Plant Based Diets
PD	Palaeolithic Diet
ROS	Reactive Oxygen Species
SBP	Systolic Blood Pressure
SFA	Saturated Fatty Acids
TDM	Traditional Mediterranean Diet
TG	Triglycerides
TNF-α	Tumor Necrosis Factor Alfa
